# Impact of Electrostatic Disorder on Intramolecular Electronic Coupling in Organic Mixed Ionic–Electronic Conductors: A Combined GRRM, MD, and QM/MM-CDFT Study

**DOI:** 10.3390/molecules31050774

**Published:** 2026-02-25

**Authors:** Zhanglei Gao, Bowen Xiao, Naoki Kishimoto, Takahiro Murashima

**Affiliations:** 1 Department of Chemistry, Graduate School of Science, Tohoku University, Aramaki, Aoba-ku, Sendai 980-8578, Japan; gao.zhanglei.p2@dc.tohoku.ac.jp (Z.G.); xiao.bowen.q7@dc.tohoku.ac.jp (B.X.); 2 Institute for Excellence in Higher Education, Tohoku University, 41 Kawauchi, Aoba-ku, Sendai 980-8576, Japan; 3 Department of Physics, Graduate School of Science, Tohoku University, 6-3 Aramaki Aza-Aoba, Aoba-ku, Sendai 980-8578, Japan; murasima@tohoku.ac.jp

**Keywords:** organic mixed ionic–electronic conductors (OMIECs), intrachain charge transport, electronic coupling, multi-scale simulation, electrostatic disorder, QM/MM, molecular dynamics

## Abstract

Organic mixed ionic–electronic conductors (OMIECs) are pivotal for bioelectronics; however, the microscopic origins of doping-dependent charge transport remain elusive. In this study, we established a multi-scale computational framework to quantify the distinct intramolecular electronic coupling ($H_{ab}$) distributions in systems with 25% and 75% doping levels. Our protocol employs automated quantum chemical calculations to exhaustively identify intrinsic local minima, ensuring thermodynamically stable initial conformations. Subsequent Molecular Dynamics (MD) simulations characterize the equilibration timescales and counter-ion dispersion behaviors. The simulation results reveal that the 75% doped system exhibits significantly stronger counter-ion confinement and a distinct electrostatic landscape compared to the 25% system. Finally, hybrid QM/MM calculations integrated with Constrained Density Functional Theory (CDFT) were utilized to evaluate $H_{ab}$ within these specific environments. The computed coupling distributions show a clear correlation with local electrostatic fluctuations induced by differing counter-ion arrangements. These findings indicate that doping-induced environmental disorder is a critical factor modulating intramolecular transport efficiency, providing a theoretical basis for optimizing OMIEC performance through electrostatic engineering.

## 1. Introduction

The seamless integration of biological systems with electronic devices represents one of the grand challenges of modern materials science, requiring interfaces that can bridge the fundamental mismatch between ionic biological signals and electronic hardware. Organic mixed ionic–electronic conductors (OMIECs) have established themselves as ideal candidates for this task due to their unique ability to transduce ionic signals—the “native language” of biology—into electronic currents with high efficiency [1,2,3]. Unlike traditional inorganic electrodes restricted to surface-limited interactions, OMIECs leverage a volumetric transduction mechanism. This allows ions to penetrate the bulk of the material, decoupling the electrochemical capacitance from the geometric footprint and enabling superior signal-to-noise ratios even at micro-scales. While initially celebrated for their pivotal roles in energy storage (e.g., nanostructured conducting polymers) [4,5] and fundamental bio-sensing [6,7,8,9], the application horizon of OMIECs is rapidly expanding in the era of artificial intelligence. Owing to their ability to continuously modulate conductance through ion intercalation, OMIECs are now positioned at the forefront of neuromorphic computing. Devices such as Organic Electrochemical Transistors (OECTs) and ionic floating-gate memories enable artificial synapses that simulate biological plasticity (e.g., LTP/LTD) with high linearity, low noise, and low energy consumption, offering a promising pathway toward efficient in-memory computing architectures [10,11,12,13,14,15,16,17]. Furthermore, as the demand for high-bandwidth neural communication grows, OMIECs have become indispensable candidates for next-generation brain–computer interfaces (BCIs) [15,18]. Their mechanical compliance and superior mixed conductivity effectively resolve the critical mechanical and impedance mismatch between rigid silicon electronics and soft neural tissues, paving the way for long-term, minimally invasive neural integration [18,19].

Central to these emerging applications is the fundamental operation of electrochemical doping, a process where ions from an electrolyte are injected into the organic semiconductor channel to compensate for the electronic charge generated upon oxidation or reduction. The efficiency of this process is governed by the ability of the polymer backbone to accommodate charge carriers (polarons or bipolarons) stabilized by penetrating counter-ions. This dynamic mechanism effectively modulates the bulk conductivity of the material, allowing it to switch between insulating and conducting states. Consequently, the performance of OMIEC devices is intrinsically linked to the polymer morphology and the complex local electrostatic environment, which jointly dictate the efficiency of mixed ionic and electronic transport. To optimize this, recent research has heavily focused on synthetic strategies to tailor molecular packing and microstructural order. For instance, side-chain engineering—specifically adjusting hydrophilicity and chain length—has been shown to significantly influence the lamellar stacking distances and the degree of water uptake [20]. However, this introduces a complex trade-off: while hydrophilic side chains facilitate ionic mobility, excessive swelling may disrupt the π−π stacking required for electronic connectivity. Additionally, mechanisms such as ion-pair absorption can enhance device speed by facilitating charge compensation [21]. The operation is further complicated by the fact that electrochemical doping induces dynamic, reversible changes in crystallinity and microstructure [22,23,24,25], and material selection (e.g., n-type vs. p-type) remains critical for complementary circuits [26,27,28]. Understanding the fundamental mass and charge transport dynamics within these disordered, fluctuating systems is therefore crucial [29].

Despite these advances, a microscopic understanding of charge transport remains elusive. While detailed descriptions of mass and charge transport dynamics have been attempted [29,30], the complex interplay between molecular conformation and the ionic environment is often oversimplified. Recently, pioneering works by Troisi et al. [31,32] have fundamentally reshaped this view by demonstrating that the electrostatic disorder in OMIECs is highly dynamical. Specifically, they revealed that the excess charge density fluctuates significantly during Molecular Dynamics (MD) simulations [31] and that the site energies (diagonal disorder) exhibit broad, environment-dependent distributions [32].

However, while these studies provided a groundbreaking characterization of the energetic landscape, two critical gaps remain. First, previous studies typically rely on standard MD equilibration from random packing, which may remain trapped in metastable conformational states due to the high degrees of freedom in OEG-side-chain polymers. Our approach addresses this by employing Global Reaction Route Mapping (GRRM) to exhaustively identify low-energy local minima, ensuring that our subsequent simulations are anchored to the most energetically representative structural baseline. Second, while the “depth” of energetic traps (diagonal disorder) is now better understood, the dynamic variability of the intrachain electronic coupling ($H_{ab}$)—the “off-diagonal” bridge facilitating charge transfer between specific adjacent units—remains to be quantified under realistic, fluctuating environments. By systematically analyzing fixed pairs of thiophene rings within the p(g2T-T) backbone, we aim to disclose how the surrounding hydrated ions modulate the connectivity of the transport network, providing the missing off-diagonal component to the current understanding of dynamic disorder.

**Figure 1:**
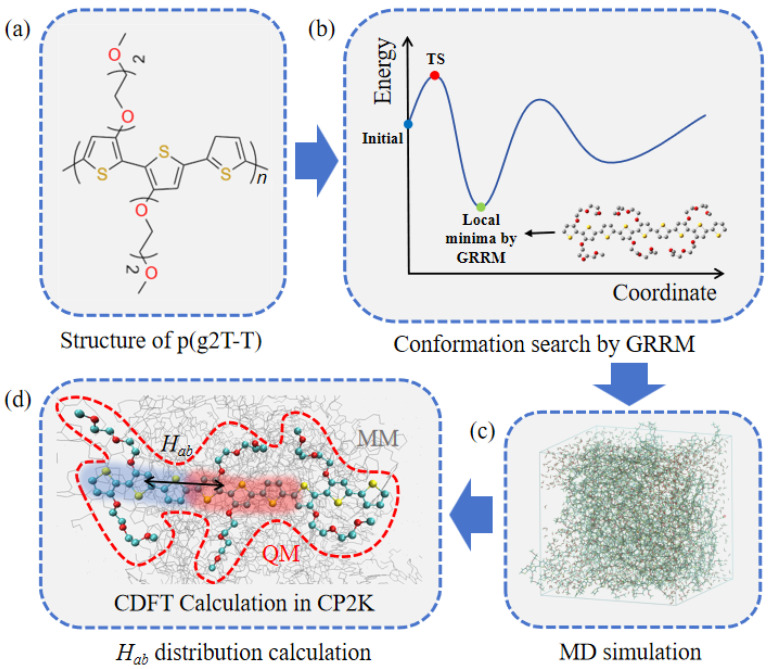
Schematic illustration of the multi-scale computational framework adopted in this study. (**a**) Chemical structure of the p(g2T-T) polymer (where *n* = 3 corresponds to the trimer model used as the fundamental unit in this study) (**b**) Systematic conformation search and potential energy surface (PES) exploration using the Global Reaction Route Mapping (GRRM) method. (**c**) Molecular Dynamics (MD) simulations performed on amorphous cells to sample bulk morphology and dynamic disorder. (**d**) Electronic coupling (*H_ab_*) calculation using a hybrid QM/MM approach integrated with Constrained Density Functional Theory (CDFT), where the central trimer is treated at the QM level (highlighted) and the environment at the MM level.

To bridge this gap, we present a comprehensive multi-scale computational framework, schematically illustrated in Figure 1, which adopts a “bottom-up” strategy to systematically decouple the intricate factors governing charge transport. The workflow begins with the definition of the fundamental chemical model: a p(g2T-T) trimer (Figure 1a). This specific oligomer, comprising alternating glycolated bithiophene (g2T) and thiophene (T) units, was selected to balance computational feasibility with the necessity of mitigating finite-size edge effects, ensuring a representative electronic environment for the central unit.

To establish a rigorous structural baseline, we employ Global Reaction Route Mapping (GRRM) to explore the potential energy surface (PES), as shown in Figure 1b. Unlike conventional stochastic sampling techniques, GRRM provides a systematic and exhaustive search for all intrinsic local minima and transition states (TS) on the PES [33,34,35]. First, recognizing the disparate flexibility between the rigid conjugated backbone and flexible side chains, we perform manual screening to locate initial side-chain minima, thereby avoiding computational waste on trivial rotations. Second, the Anharmonic Downward Distortion Following (ADDF) method is applied to exhaustively map the PES. This step is essential for establishing a chemically meaningful baseline structure, particularly in conjugated polymers, where backbone planarity significantly influences orbital overlap and charge delocalization.

Building upon these identified conformers, we perform Molecular Dynamics (MD) simulations within a periodic amorphous cell (Figure 1c). This step samples the realistic bulk morphology, capturing the “dynamic disorder” of entangled polymer chains interacting with water and ions at varying oxidation states [36,37]. These simulations reveal how charge state-dependent ion condensation modulates local dielectric environments and induces structural distortions—phenomena that cannot be captured by static DFT calculations alone.

Finally, to quantify the intrachain charge transport efficiency, we extract snapshots from the MD trajectories for hybrid QM/MM calculations integrated with Constrained Density Functional Theory (CDFT) (Figure 1d) [38,39]. In this setup, the central trimer is treated quantum mechanically (QM region), while the surrounding polymer chains and solvent environment are modeled as a classical background (MM region, depicted as gray wires in Figure 1d). CDFT allows for the precise definition of diabatic charge-localized states (schematically illustrated as overlapping red/blue orbitals), enabling the direct computation of the electronic coupling ($H_{ab}$) matrix element [38,39]. Crucially, because these calculations are performed across thermally sampled configurations, the resulting distribution of $H_{ab}$ values reflects the statistical impact of environmental heterogeneity and conformational dynamics on transport properties.

## 2. Results

### 2.1. Conformational Energy Landscape and Global Minimum Structure

**Figure 2:**
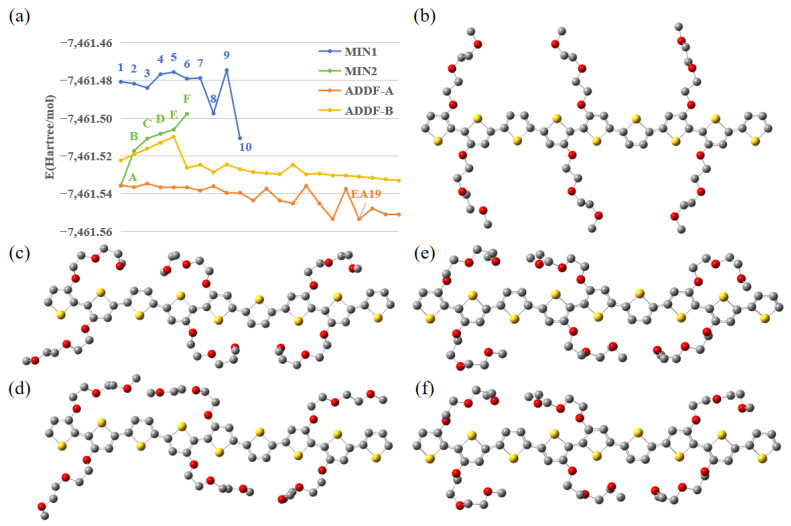
(**a**) Energy profiles of the hierarchical conformational search plotted against the conformer index (n), including coarse screening (MIN1, blue), fine refinement (MIN2, green), and exhaustive ADDF exploration (orange/yellow). (**b**) The lowest energy conformation (E11) obtained from MIN1. (**c**) The optimal conformation A and (**d**) the sub-optimal conformation B obtained from MIN2. (**e**) The EA19 (neutral state) identified via ADDF. (**f**) The optimized cationic structure derived from EA19. Grey, red, and yellow spheres represent carbon, oxygen, and sulfur atoms, respectively.

The thermodynamic ground state of the p(g2T-T) trimer was investigated using the GRRM protocol [33,35]. Figure 2a illustrates the energy trajectory of the system throughout the hierarchical screening process. In the first round of coarse screening (MIN1, blue trajectory), ten distinct initial conformations were optimized. Among them, Conformer 10 exhibited significantly lower energy compared to the others (Points 1–9), establishing it as the most stable candidate from this initial set. Proceeding to the second round of fine refinement (MIN2, green trajectory), we explored variations of Conformer 10, yielding conformers A through F. Here, Conformer A was identified as the energy minimum, followed by Conformer B as a stable secondary minimum. Finally, the ADDF search phase (orange trajectory), initiated from both A and B, traversed multiple potential energy barriers—characterized by distinct “sawtooth” peaks—and descended into a deeper energy basin. This exhaustive process identified Conformer EA19 (derived from A) as the most stable conformer, which possesses a total energy significantly lower than any precursor found in the manual screening phases.

Structurally, the initial structure E11 (Figure 2b) and intermediate conformers A and B (Figure 2c,d) displayed “splayed” or semi-extended side-chain orientations. In contrast, the most stable conformer identified (EA19; Figure 2e) exhibited a distinct morphology where specific oligoethylene glycol side chains were folded inward, occupying the intramolecular voids along the conjugated backbone. Upon optimization in the cationic state (+1 charge), the EA19 geometry (Figure 2f) retained its overall coordinates and the inward-folded side-chain configuration with minimal structural deviation compared to the neutral state.

### 2.2. Dynamic Equilibration and Microscopic Morphology

The thermodynamic stability and structural evolution of the amorphous p(g2T-T) systems at 25% and 75% doping levels were monitored over a 40 ns MD trajectory.

#### 2.2.1. Thermodynamic Stability and Component Dynamics

**Figure 3:**
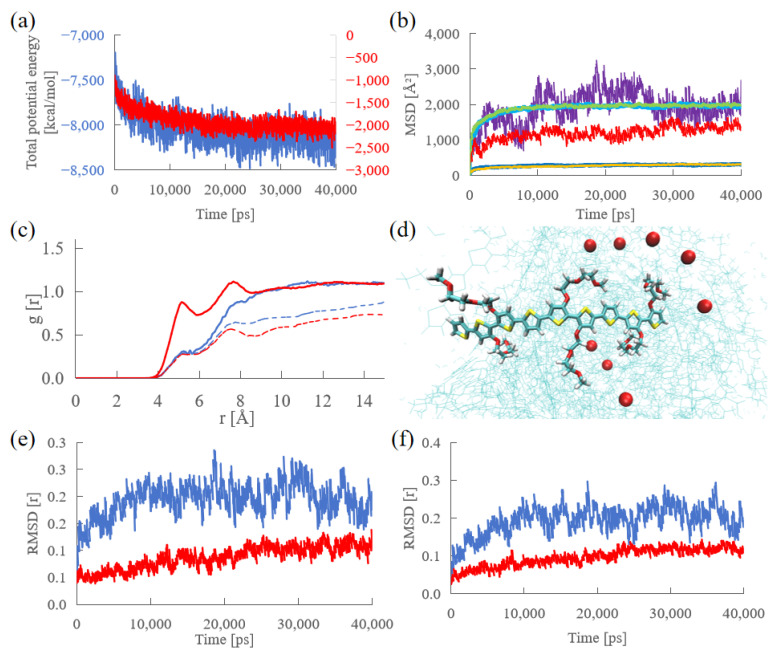
Evolution of thermodynamic and structural properties during the 40 ns MD production runs for 25% and 75% doping systems. (**a**) Total potential energy convergence (Blue: 25% system; Red: 75% system). (**b**) Mean Squared Displacement (MSD) of system components. For the 25% system, Cl^−^, polymer, and water are represented by purple, blue, and light blue lines, respectively. For the 75% system, the corresponding components are shown in red, yellow, and green, respectively. (**c**) RDF of Cl^−^ around side-chain oxygen atoms. Solid lines represent interactions with cationic units, and dashed lines represent interactions with neutral units (Blue lines: 25% system; Red lines: 75% system). (**d**) Representative snapshot of the 75% doping system, visualizing the dense distribution of Cl^−^ ions (red spheres) around the cationic backbone. (**e**,**f**) Time-dependent RMSD of Cl^−^ RDF profiles relative to the backbone C and side-chain O atoms (Blue: 25%; Red: 75%).

As shown in Figure 3a, the total potential energy for both systems showed a rapid decay initially and reached a steady asymptotic plateau after approximately 20 ns. This indicates the effective elimination of high-energy steric contacts present in the initial random packing. The Mean Squared Displacement (MSD) analysis (Figure 3b) indicated that water molecules and polymer chains exhibited similar diffusive behaviors in both systems. However, the chloride (Cl^−^) counter-ions showed distinct differences: in the 25% system, Cl^−^ ions displayed higher mobility with larger fluctuations, whereas in the 75% system, the Cl^−^ MSD profile was significantly suppressed and relatively flat. This behavior aligns with observations of charge state-dependent ion condensation in conjugated polymers [36] and is consistent with the nanosecond-scale ion dynamics reported by Troisi et al. [31] for the p(g2T-T) system, where increased doping levels lead to a more constrained environment for anions.

#### 2.2.2. Structural Analysis and Convergence Verification

The Radial Distribution Function (RDF) between Cl^−^ and the backbone oxygen atoms (Figure 3c) exhibited two distinct, high-intensity peaks located at approximately 5 Å and 7 Å in the 75% system. This trend of charge-dependent ion localization—where anions exhibit significantly stronger and closer coordination with the cationic backbone compared to the neutral or low-doped states—closely matches the radial distribution profiles observed by Troisi et al. [31] for the same polymer/electrolyte interface. This trend was consistently observed in other key interaction pairs. As shown in Figure S1 (Supplementary Materials), the distribution profiles of Cl^−^ relative to the polymer backbone carbons (main_C) and thiophene sulfurs (S) further corroborated that counter-ions in the 75% system are more tightly bound to the conjugated backbone compared to the 25% system. In the 25% system, the corresponding peak intensity was lower. Simulation snapshots (Figure 3d) for the high-doping system visualized multiple Cl^−^ ions located in the immediate vicinity of the polymer backbone. The time-dependent RMSD profiles of the Cl^−^ distribution relative to the polymer backbone and side chains (Figure 3e,f) showed that while potential energy stabilized early, the structural arrangement continued to evolve until approximately 30 ns. In the 30–40 ns window, the RMSD values oscillated around a horizontal baseline, confirming the equilibrium of the overall component distribution, particularly the delocalization and localization patterns of chloride ions. To further confirm the global equilibrium of the simulation box, the convergence of hydration shells (backbone C—water O) and specific ion–polymer coordination (S—Cl^−^) was also examined. As shown in Figure S2 (Supplementary Materials), these interactions exhibited similar convergence behaviors, oscillating around a stable baseline after 30 ns. Consequently, to ensure maximum reliability for electronic structure analysis, the simulation was extended for an additional 10 ns beyond the initial 40 ns trajectory. All snapshots used for subsequent calculations were rigorously extracted from this post-40 ns interval.

### 2.3. Electronic Coupling Distribution Under Electrostatic Disorder

Based on the snapshots extracted at 0.5 ns intervals from the additional 10 ns post-equilibration trajectory (post-40 ns), we quantified the intrachain electronic coupling ($H_{ab}$) to link microscopic structure to charge transport efficiency.

#### 2.3.1. Coupling Distribution and Energetic Disorder

**Figure 4:**
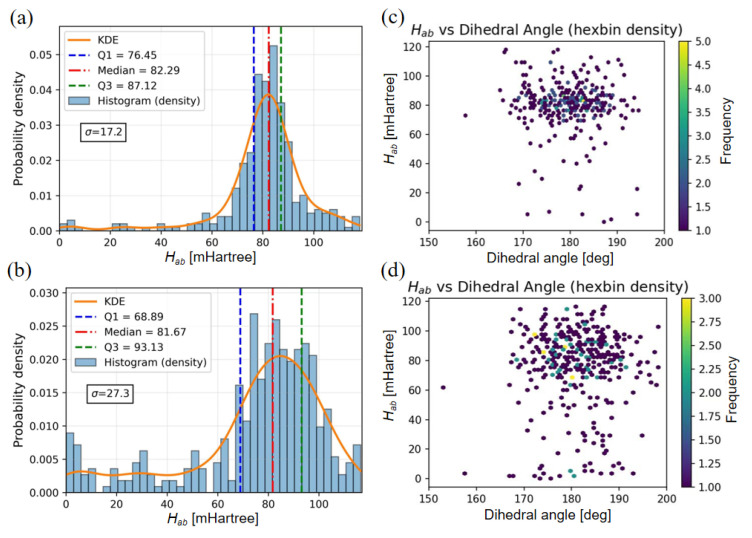
Statistical analysis of electronic couplings ($H_{ab}$) and their geometric correlations in different doping systems. (**a**,**b**) Probability density distributions of $H_{ab}$ for the (**a**) 25% and (**b**) 75% doping systems. The histograms (blue bars) are overlaid with Kernel Density Estimation (KDE) curves (orange lines), and the standard deviation (σ) is annotated to quantify energetic disorder. (**c**,**d**) Hexbin density plots correlating $H_{ab}$ with the backbone dihedral angle for the (**c**) 25% and (**d**) 75% systems. The color scale (labeled ‘Frequency’) quantifies the population density of sampled conformations within each hexagonal bin, visualizing the joint probability of observing specific coupling-dihedral configurations.

Figure 4a,b illustrate the normalized histograms (bars) and kernel density estimates (curves) of the electronic coupling values ($H_{ab}$) for the 25% and 75% doping systems, respectively. While the distributions in both systems are centered around similar median values (~82 mHartree), their breadths exhibit a marked difference. The standard deviation (σ) for the 25% system is 17.2 mHartree, whereas it increases significantly to 27.3 mHartree for the 75% system (Figure 4a vs. Figure 4b). This distinct broadening in the highly doped regime points to a higher degree of energetic disorder, suggesting that charge carriers in the 75% system navigate a significantly more heterogeneous electrostatic landscape compared to the lightly doped system.

#### 2.3.2. Geometric Correlations

To decouple intrinsic geometric effects from environmental influences, we examined the correlation between $H_{ab}$ and the backbone dihedral angle between donor and acceptor units, as shown in Figure 4c (25%) and Figure 4d (75%). In both systems, the data points cluster densely within the near-planar region (175–185°), confirming that the backbone planarity predicted by GRRM is largely preserved in the bulk phase regardless of doping level. However, a comparison of the vertical spread in Figure 4c,d reveals that, despite similar geometric configurations, the coupling values fluctuate much more dramatically in the 75% system. This implies that the observed energetic disorder is driven less by backbone torsional defects and more by extrinsic factors [40].

#### 2.3.3. Impact of Local Ionic Environment

To rigorously assess how the local environment influences electronic coupling, we quantified the ionic crowding by counting the Chloride Coordination Number ($N_{Cl}$) within a 10 Å spherical cutoff around the donor–acceptor pair. We then computed the conditional probability $P(H_{ab}<Q_{1}|N_{Cl}=k)$, representing the likelihood that a sample’s coupling value falls below the first quartile threshold ($Q_{1}$) given a specific coordination number k.

**Figure 5:**
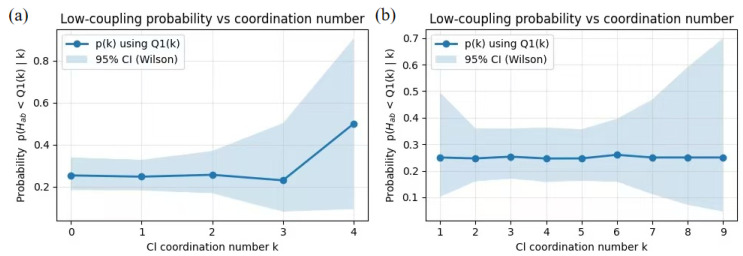
Statistical correlation between local ionic environment and electronic coupling states. (**a**,**b**) Conditional probability $P(H_{ab}<Q_{1}|N_{Cl}=k)$ of observing a low-coupling state (below the first quartile threshold, $Q_{1}$) as a function of the chloride coordination number $(N_{Cl}$) for the (**a**) 25% and (**b**) 75% doping systems. $N_{Cl}$ is defined as the number of chloride ions within a 10 Å spherical radius of the coupling segment. The shaded areas represent 95% Wilson confidence intervals.

As shown in Figure 5a, the 25% system exhibits a tentative upward trend in probability at higher coordination numbers (k=4), suggesting that the formation of dense local ion clusters in the lightly doped regime can perturb wavefunction overlap and increase the propensity for low-coupling states. In contrast, the 75% system (Figure 5b) displays a remarkably flat probability profile across a broad range of coordination numbers (k=1−9). This indicates that in the highly doped regime, the electrostatic landscape is uniformly crowded; consequently, marginal variations in local coordination do not significantly alter the likelihood of observing weak electronic coupling, pointing to a saturation of environmental screening effects.

## 3. Discussion

### 3.1. Mechanisms of Conformational Stabilization

The identification of EA19 as the most stable conformer highlights the critical role of non-covalent interactions in determining the backbone planarity and energetic stability of OMIECs. The morphological transition from “splayed” side chains in high-energy conformers to the “self-wrapping” motif in EA19 suggests that intramolecular dispersion forces and weak hydrogen bonds (C–H···O) are the primary drivers for stabilization [20]. By tucking into the backbone voids, the side chains minimize the solvent-accessible surface area and mechanically “lock” the conjugated core. Importantly, the structural robustness observed upon oxidation implies that this self-wrapped conformation resides in a deep potential well, making it a thermodynamically resilient scaffold that persists even under the electrostatic perturbations of doping. This validates EA19 as a chemically meaningful starting point for bulk simulations, superior to arbitrarily constructed linear chains.

### 3.2. Doping-Induced Dynamic Disorder and Ion Confinement

The MD results reveal that the microscopic environment of charge carriers is strongly modulated by the doping level. The distinct behavior of Cl^−^ ions in the MSD analysis points to a cooperative electrostatic effect [36]. In the 25% system, the sparse charge centers result in a weak binding regime, allowing ions to diffuse via a hopping mechanism. Conversely, at 75% doping, the high density of cationic sites along the backbone creates a continuous, deep electrostatic potential well. This leads to the electrostatic confinement (or trapping) of counter-ions [21,37], as evidenced by the flattened MSD and the sharp RDF peaks.

This confinement has profound implications for electronic properties. The formation of a tight, persistent ion sheath (as seen in Figure 3d) introduces a strong, inhomogeneous electric field directly acting on the conjugated backbone. Unlike the fluctuating, diffuse environment in the low-doping regime, the high-doping environment is characterized by a more rigid and intense electrostatic landscape. This difference in the local ionic environment—specifically the transition from diffuse ion clouds to tight ion pairing—is expected to be the key factor differentiating the charge transport efficiencies between the two doping regimes.

### 3.3. Statistical Origins of Energetic Disorder

The CDFT analysis provides a detailed breakdown of how microscopic fluctuations translate into the energetic disorder governing charge transport. Comparing the normalized histograms and kernel density estimates reveals a fundamental divergence in disorder mechanisms. The 25% system exhibits a relatively narrow electronic coupling distribution with an Interquartile Range (IQR) of 10 mHartree and a median of 82.29 mHartree. The weak long-tail contribution suggests that coupling variations in the low-doping regime primarily arise from rapid, intrinsic structural and electrostatic fluctuations within the chain, while static conformation-driven disorder remains minor. This is consistent with the absence of extremely small values that would typically indicate severe torsional breaks.

In contrast, the 75% system displays a significantly broadened distribution with an IQR of 15 mHartree and a median of 81.67 mHartree, accompanied by a pronounced long-tail population. This increased width reflects a higher degree of structural and electrostatic variability at elevated doping levels. Notably, the similar median values between the two systems suggest that the predominant backbone conformations preserve their intrinsic conjugation potential regardless of doping density. However, the substantial tail population and the presence of secondary maxima in the 75% system point to the emergence of multiple metastable conformational or environmental states.

The scatter heatmaps of coupling values versus dihedral angles further clarify the source of this disorder. In the 75% system, the data is scattered across a wide range, indicating that the relationship between the dihedral angle and coupling strength is no longer monotonic. Even within the main near-planar conformational group, point-like scatter is observed where low coupling values (<30 mHartree) appear despite favorable geometries. This demonstrates that static disorder does not arise solely from conformational distortions; rather, doping-induced environmental fluctuations contribute significantly to the disruption of orbital overlap. Conversely, the 25% system remains much less dispersed, with rare occurrences of low coupling values, indicating that dynamic disorder remains the dominant source of variability.

The analysis of low-coupling probability as a function of chloride coordination reveals no systematic increase or decrease across coordination states in the highly doped system. The substantial overlap of confidence intervals indicates that the occurrence of low-coupling events is statistically independent of the simple number of coordinated ions. This implies that energetic disorder in the high-doping regime is not driven by a simple ion-counting metric but by more complex environmental factors—such as ion spatial distribution, local electric-field heterogeneity, and packing fluctuations [8,29]. In this saturated regime, the specific arrangement of the ionic sheath, rather than its density, governs the formation of charge traps.

It is instructive to contextualize these findings within the emerging understanding of dynamic disorder in OMIECs. Our observations of the fluctuating electrostatic environment are in excellent agreement with the recent pioneering works by Troisi et al. [31,32]. They established that the motion of water and ions creates a highly dynamic landscape for both the excess charge distribution [31] and the site energies (diagonal disorder) [32], concluding that static models are insufficient to capture the physics of OMIECs. Our study complements and extends these findings by revealing that this dynamic disorder also profoundly impacts the off-diagonal component of charge transport—the intrachain electronic coupling ($H_{ab}$). While Troisi et al. demonstrated that the “depth” of the energetic traps fluctuates with time, our results show that the “width” of the bridges (coupling) between monomer units is equally susceptible to environmental modulation. Specifically, we observe that even for a fixed backbone conformation, the rearrangement of ions can induce significant variations in $H_{ab}$. This implies that the “dynamic disorder” in OMIECs is all-encompassing, simultaneously modulating both the energy of the charge carriers and the efficiency of their hopping pathways.

While chloride (Cl^−^) was utilized as a model counter-ion in this study, the observed trends of electrostatic localization and coupling modulation are expected to be qualitatively representative of OMIEC systems doped with other monovalent anions. However, it should be noted that variations in ion size, valence, and solvation energy could further refine the electrostatic landscape. As demonstrated in studies of poly(ionic liquid) systems [41], the specific structure and bulkiness of anions (such as TFSI^−^ versus $PF_{6}^{-}$) significantly influence the local interaction environment, interfacial polarization, and the resulting counterion mobility. Specifically, larger anions exhibit different coordination distances and charge delocalization patterns, which may alter the intensity and spatial heterogeneity of the local electric fields acting on the backbone. Similarly, multivalent ions or ions with specific non-covalent interaction capabilities could induce stronger coulombic confinement or unique docking configurations, leading to even more pronounced energetic disorder [42]. Our findings provide a foundational framework, and future studies incorporating a broader range of ionic species will be essential to fully generalize the impact of ion–polymer interactions on charge transport.

Qualitatively, the increased energetic disorder (both diagonal and off-diagonal) observed at high doping levels is expected to hinder macroscopic charge transport. While high carrier density is generally beneficial for conductivity, our results suggest that the formation of deep electrostatic traps and the disruption of electronic coupling pathways in the 75% system may lead to severe carrier localization. This provides a microscopic rationale for the experimentally observed saturation or decrease in mobility often reported in OMIECs at high oxidation levels [2,43].

### 3.4. Limitations of the Methodology

While our multi-scale approach effectively captures the dynamic nature of electronic coupling, certain limitations should be noted.

First, our calculations focused on the intrachain electronic coupling between thiophene rings, effectively capturing the backbone transport potential. However, macroscopic charge transport in OMIEC materials is a three-dimensional process that relies heavily on interchain hopping pathways. Theoretically, macroscopic mobility (μ) could be derived by calculating charge transfer rates ($k_{ET}$) via Marcus theory and propagating them through Kinetic Monte Carlo (KMC) simulations [44,45]. In such a framework, the significant variance in $H_{ab}$ distributions observed in our study (particularly in the 75% system) implies a broad distribution of hopping probabilities. Since the charge transfer rate typically scales quadratically with the electronic coupling ($k_{ET}\propto |H_{ab}{|}^{2}$) in the non-adiabatic regime, the observed fluctuations in $H_{ab}$ would translate into substantial variations in local hopping rates. This pronounced off-diagonal disorder is expected to propagate into the KMC dynamics, potentially leading to dispersive transport characteristics where carriers exhibit a heavy-tailed distribution of waiting times, thereby limiting the effective macroscopic mobility [44]. However, the rigorous QM/MM-CDFT method employed here is computationally intensive, requiring significant resources per data point to maintain ab initio accuracy. Consequently, generating the massive dataset of intermolecular coupling pairs required for statistically significant KMC predictions is currently prohibitive on standard computing servers. While this limits direct quantitative comparison with experimental mobility in this specific study, our results provide a high-precision baseline for future work that aims to integrate more efficient coupling estimation methods to bridge this gap.

Second, the classical MD simulations employ non-polarizable force fields, which may underestimate screening effects in highly polarizable conjugated backbones.

Third, our current MD simulations utilized a static charge model for the doped oligomers, whereas previous work [31] has demonstrated that the excess charge on the polymer backbone undergoes dynamic rearrangement over nanosecond timescales in response to the fluctuating environment. Although our CDFT calculations capture the instantaneous impact of counter-ion distribution on $H_{ab}$ via individual snapshots, the absence of a self-consistent charge-update scheme during the MD propagation—similar to the methodology adopted in [31]—may introduce slight deviations in the sampled ion–polymer coordination statistics. Accounting for such dynamic charge redistribution would likely yield a more precise description of the coupled ionic–electronic landscape. Future work will aim to extend this framework to longer oligomers, incorporate polarizable models, and explore efficient algorithms for intermolecular coupling to further refine the description of the electrostatic landscape and macroscopic transport properties.

## 4. Materials and Methods

### 4.1. Overview of Computational Materials

The computational investigation employed a suite of specialized software packages to address different spatial and temporal scales. Global Reaction Route Mapping (GRRM17), coupled with Gaussian 16 (Gaussian, Inc., Wallingford, CT, USA), was used for potential energy surface exploration [46]. Classical Molecular Dynamics (MD) simulations were performed using the LAMMPS software (version 29 Aug 2024) package [47]. For electronic structure and coupling calculations, we utilized the hybrid QM/MM module within CP2K/Quickstep (version 2025.1) [48]. The primary material model, the p(g2T-T) trimer (Figure 1a), was constructed with three conjugated repeat units and six ethylene glycol (EG2) side chains to serve as a representative oligomer for bulk simulations.

### 4.2. Conformational Space Exploration via GRRM

To establish a stringent thermodynamic baseline for the p(g2T-T) system, we utilized the GRRM strategy to exhaustively explore the potential energy surface (PES) [49,50,51,52]. Unlike stochastic sampling methods, GRRM is an automated technique that identifies intrinsic local minima (EQ) and transition states (TS), which are particularly critical for accurately identifying the most stable conformer and understanding reaction pathways in complex conjugated systems [49,53,54,55].

#### 4.2.1. Model Definition and Simplification

To balance computational feasibility with the need to capture side-chain dynamics, we constructed a simplified p(g2T-T) trimer model [54,56]. The conjugated backbone was maintained at three repeat units to preserve electronic characteristics. Crucially, each repeat unit bears two oligoethylene glycol side chains, resulting in a total of six side chains for the trimer. To manage computational cost while retaining conformational flexibility, each side chain was truncated to two ethylene glycol repeat units (EG2).

#### 4.2.2. Hierarchical Search Protocol

We adopted a multi-stage screening protocol combining manual chemical intuition with automated algorithms [57]. First, during the Coarse Manual Screening stage, we initially constructed 10 distinct starting conformations based on the primary orientation vectors of the side chains relative to the backbone. Minimum point optimization (MIN) calculations were performed to screen these initial candidates and identify the energetically most favorable structure to serve as the baseline for further refinement. Subsequently, in the Fine Structural Refinement stage, we further refined the conformational space around the identified candidate by performing a secondary screening. This step focused on the specific bending (curved vs. extended) and planar orientation (in-plane vs. out-of-plane, viewer-facing vs. away-facing) of the six side chains, generating multiple subclass variants for subsequent optimization. Finally, we conducted Exhaustive Exploration via ADDF. To ensure no low-lying energy states were missed, we utilized the most stable geometries identified from the screening phases as starting points for ADDF calculations [35,49,51,52]. This extensive search was employed to map the surrounding equilibrium structures (EQs) and rigorously locate the most stable conformer, confirming the robustness of the final configuration used for subsequent simulations [58].

#### 4.2.3. Computational Details

All GRRM calculations (MIN and ADDF) were executed using the GRRM17 program coupled with Gaussian 16 for energy and gradient evaluations [56]. Calculations were carried out at the B3LYP/3-21G level of theory. To account for the aqueous environment relevant to OMIEC operation, implicit solvation was included via the Polarizable Continuum Model (PCM) with water as the solvent (SCRF = (PCM, Solvent = Water)) [59]. Specific parameters for the ADDF search were set as follows: The search followed the 15 lowest local minima (NLowest = 15) using the Large ADDF algorithm (LADD = 3). The search was focused on identifying equilibrium structures (EQOnly) at a temperature of 300.0 K. Dissociation criteria were set with UpDC = 10 and DownDC = 10. Gaussian processing parameters were set to GauProc = 30 and GauMem = 5000.

### 4.3. Molecular Dynamics Simulations and Amorphous Morphology Generation

To bridge the gap between single-chain quantum mechanics and macroscopic material properties, classical MD simulations were performed using the LAMMPS software package to construct representative bulk morphologies [60,61,62,63,64,65,66,67,68,69].

#### 4.3.1. Force Field Parameterization

The most stable conformer identified (EA19), obtained from the GRRM search, served as the fundamental building block [49,50,51,52]. The p(g2T-T) trimer was selected as the fundamental computational unit for the electronic coupling analysis. Since the intrachain electronic coupling ($H_{ab}$) is a local property primarily governed by the orbital overlap between nearest-neighbor monomer units, a trimer provides two complete inter-ring junctions. This length is sufficient to capture the essential local conformational flexibility and the screening effects of the surrounding electrostatic environment explicitly treated in our QM/MM scheme, without the prohibitive computational cost associated with longer oligomers. Regarding the terminal effects, although the trimer features asymmetric ends (with distinct glycol-substituted and unsubstituted thiophene termini), our QM/MM-CDFT analysis focuses specifically on the electronic coupling between the central ring pairs. The terminal units primarily serve as electronic boundary conditions to mitigate finite-size edge effects, ensuring that the wavefunction overlap in the region of interest mimics the bulk intrachain environment. To ensure seamless compatibility with the subsequent QM/MM calculations in CP2K, the bonded and non-bonded interactions of the p(g2T-T) trimer were described by the General Amber Force Field (GAFF) [69]. To accurately capture the electrostatic potential of the conjugated backbone, atomic partial charges were derived using the Restrained Electrostatic Potential (RESP) fitting method based on the DFT-calculated electron density at the B3LYP/6-311G(d,p) level [70,71].

#### 4.3.2. System Construction and Doping Protocol

To simulate the realistic electrochemical environment, amorphous simulation cells were constructed using the Packmol algorithm [62]. Specifically, 64 p(g2T-T) trimers were randomly packed into a cubic box (*L* = 55.53 Å) to form a continuous, entangled amorphous phase with an initial density of $1.2\;{g/cm}^{3}$. This aggregated polymer network, referred to hereafter as the polymer matrix, was solvated with 1624 water molecules modeled by the SPC/E potential.

To investigate the impact of oxidation states on morphology and charge transport, we constructed systems at two distinct doping levels: 25% and 75%. Following the computational framework established by Troisi et al. [31], we selected these specific doping levels to ensure direct comparability with previous studies on charge distribution. These levels also correspond to two critical and experimentally relevant regimes in OECT operation: the 25% doping level represents the light-doping or threshold regime (onset of conductivity), while the 75% doping level corresponds to the high-oxidation or saturation regime typically encountered at high gate voltages, where ion–polymer interactions are most pronounced [2]. Specifically, in the simulation cell containing 64 oligomers, the 25% doping level was established by assigning a cationic state (+1e charge) to 16 randomly selected oligomers, neutralized by the addition of 16 chloride (Cl^−^) counter-ions. Similarly, the 75% doping level was realized by converting 48 oligomers to their cationic forms, balanced by 48 chloride counter-ions. The oxidation state was modeled by modifying the partial charges of the polymer backbone [61,64,68].

#### 4.3.3. Simulation Protocol

Following initial energy minimization, the systems were equilibrated in the isothermal–isobaric (NPT) ensemble using the Nosé–Hoover thermostat and barostat. The temperature and pressure were maintained at 300 K and 1 atm, respectively, with a time step of 1.0 fs. Crucially, despite the higher ionic concentration in the highly doped system, the equilibrated densities of the two systems remained comparable ($\rho_{25\%}\approx 1.21\;{g/cm}^{3}\;and\;\rho_{75\%}\approx 1.20\;{g/cm}^{3}$). This consistency ensures that any observed variations in dynamic properties (e.g., MSD) originate from the electronic interactions and oxidation states rather than significant changes in the system’s free volume. The production runs were then executed using a robust Python (version 3.9)-scripted loop protocol to ensure simulation stability and efficient data management [69]. The total simulation time was 40 ns, divided into 2000 cycles of 20 ps each. At the end of each 20 ps loop, a restart data file was generated to serve as the input for the subsequent cycle.

Convergence Criteria and Analysis: To ensure that the amorphous cells reached a fully equilibrated and homogeneously dispersed state prior to electronic structure analysis, we monitored three distinct convergence criteria. First, the total potential energy was tracked to verify thermodynamic stability. Second, the Mean Squared Displacement (MSD) was analyzed to assess the diffusive dynamics of the system components. Third, and most rigorously, we evaluated structural convergence by monitoring the time evolution of the Radial Distribution Function (RDF), g(r), defined as follows (Equation (1)):

(1) $$g(r)=\frac{dn(r)}{4\pi r^{2}dr\rho }$$

where dn(r) represents the number of particles in a spherical shell of thickness dr at a distance r, and ρ is the number density of the bulk system. Specifically, to quantify the drift in the solvation structure, we calculated the Root Mean Square Deviation (RMSD) between the RDF of the N-th cycle ($g_{N}(r)$) and that of the initial reference cycle ($g_{ref}(r)$) (Equation (2)):

(2) $$RMSD_{RDF}(N)=\sqrt{\frac{1}{M}\sum_{i=1}^{M}[g_{N}(r_{i})-g_{ref}(r_{i}){]}^{2}}$$

where M denotes the number of radial bins. By analyzing the water–polymer interaction using this metric, we determined the precise time window where the system morphology became statistically stationary [65]. Crucially, the restart data files from this equilibrated window served a dual purpose: they functioned as “snapshots” containing instantaneous molecular coordinates, which were directly extracted to serve as input structures for the subsequent electronic coupling ($H_{ab}$) calculations.

### 4.4. Electronic Coupling via Hybrid QM/MM-CDFT Calculations

The core of our methodology involves rigorously evaluating intrachain charge transport capability within the disordered electrostatic environment generated by MD [40,59,72,73,74]. This was achieved using a hybrid Quantum Mechanics/Molecular Mechanics (QM/MM) approach integrated with Constrained Density Functional Theory (CDFT), implemented in the CP2K/Quickstep package (version 2025.1) [59,72,73,75,76]. The input file structures for the CDFT calculations were adapted from templates generated by the Multiwfn wavefunction analysis program [77,78].

#### 4.4.1. Generation of AMBER Topology and Coordinates

To enable QM/MM calculations in CP2K, we established a robust workflow using the AmberTools suite to generate the required AMBER topology (.prmtop) and coordinate (.inpcrd) files [69]. First, a representative single p(g2T-T) trimer structure was extracted from the equilibrated MD trajectory (center.pdb), and force field parameters were generated using antechamber with the GAFF atom types. Partial atomic charges were assigned using the AM1-BCC method to ensure compatibility with standard AMBER protocols for organic molecules. Missing parameters were supplemented using parmchk2. Subsequently, the full snapshot containing the bulk system (e.g., 64 oligomers, counter-ions, and water) was processed using a custom Python script. This step was crucial to format the PDB file according to AMBER conventions, including assigning unique atom names within residues, inserting TER delimiters, and defining residue names (e.g., MOL for polymer, Cl- for ions, and WAT for water). Finally, the tleap program was employed to assemble the complete system topology. It loaded the custom parameters (.mol2 and .frcmod) for the polymer, along with standard libraries for water and ions, producing the final topology and coordinate files used for the QM/MM input.

#### 4.4.2. QM/MM Partitioning and Sampling Strategy

To comprehensively evaluate the impact of dynamic structural evolution on electronic coupling, we extended the MD production runs by an additional 10 ns post-equilibration [79,80]. From these trajectories, snapshots were sampled at 0.5 ns intervals to ensure statistical independence and capture dynamic coupling fluctuations. Electronic coupling calculations were performed on all charged molecules within the selected snapshots. This rigorous sampling protocol was designed to generate a statistically significant dataset for both the 25% and 75% doping systems, allowing for the construction of robust probability distributions of the coupling values. A single p(g2T-T) trimer was defined as the quantum region (QM Region, Charge +1, Multiplicity 2), ensuring the polaron wavefunction is contained within the high-level zone [81]. The surrounding environment (MM Region), including solvent molecules, ions, and adjacent polymer chains, was treated classically. The interaction between the QM and MM regions was handled using the Gaussian Expansion of the Electrostatic Potential (GEEP) scheme (ECOUPL GAUSS) with USE_GEEP_LIB 6. This approach efficiently models the electrostatic embedding, allowing the MM point charges to polarize the QM electron density [59,82].

#### 4.4.3. DFT Computational Details

The electronic structure of the QM region was solved using the Gaussian and Plane Waves (GPW) method under the Unrestricted Kohn–Sham (UKS) formalism [83,84]. We employed the PBE exchange-correlation functional [70]. Valence electrons were described by the DZVP-MOLOPT-SR-GTH basis set, while core electrons were treated using Goedecker–Teter–Hutter (GTH) pseudopotentials [70]. The auxiliary plane-wave basis set was defined with a cutoff of 300 Ry (REL_CUTOFF 50). The Self-Consistent Field (SCF) cycle utilized the Orbital Transformation (OT) method with the DIIS minimizer to ensure robust convergence (EPS_SCF 1.0E-06) [83].

#### 4.4.4. Three-Step CDFT Protocol for Electronic Coupling ($H_{ab}$)

To calculate the electronic coupling between diabatic states [72,73], we executed a hierarchical three-step workflow. As depicted in Figure 1d, the calculation focuses on the intrachain charge transfer across the central linkage connecting two adjacent repeat units, involving a total of six thiophene rings (three on the donor side and three on the acceptor side). Step 1: Optimization of State A (GACA). The excess hole (+1e) was constrained to the donor fragment (first three thiophene rings). Using Hirshfeld population analysis, a single-point energy calculation was performed to obtain the wavefunction for this localized state. Step 2: Optimization of State B (GACB). Similarly, the excess hole was constrained to the acceptor fragment (next three thiophene rings), generating the wavefunction for the second localized state. Step 3: Coupling Calculation (Mixed State). Using the restart wavefunctions from Step 1 and Step 2 as basis states, the electronic coupling matrix element $H_{ab}$ was computed via the MIXED_CDFT method as (Equation (3)):

(3) $$H_{ab}=|\langle \Psi_{A}|H|\Psi_{B}\rangle |$$

where the overlap and Hamiltonian matrices are constructed from the diabatic states. Lowdin orthogonalization (LOWDIN T) was applied to ensure proper orthonormalization of the states [26,28,85]. This rigorous protocol allows for the statistical analysis of charge transfer efficiency across the sampled dynamic disorder.

To ensure the statistical reliability of the computed electronic coupling distributions, we rigorously verified the convergence of the statistical descriptors (mean value and variance) with respect to the sample size. For the 25% doping system (16 cationic oligomers/frame), snapshots were extracted over a 10 ns window (40.0–50.0 ns), resulting in 336 samples. For the 75% doping system (48 cationic oligomers/frame), a 3.5 ns window (40.0–43.5 ns) was sufficient to yield a comparable dataset of 384 samples. As shown in Figure S3 (Supplementary Materials), both the running mean and variance of $H_{ab}$ exhibit a clear convergence trend when the accumulated sample size exceeds 200 and reach a statistically stationary state when the sample size exceeds 250. Therefore, our selected dataset sizes (>300 samples for both systems) provide a robust and conservative sampling margin to accurately capture the environmental electrostatic disorder.

## 5. Conclusions

In this study, we established a rigorous multi-scale computational framework to decipher the microscopic origins of charge transport limitations in organic mixed ionic–electronic conductors (OMIECs). By integrating Global Reaction Route Mapping (GRRM), classical Molecular Dynamics (MD), and hybrid QM/MM-CDFT calculations, we successfully decoupled the intrinsic geometric factors from extrinsic environmental influences governing the intrachain electronic coupling ($H_{ab}$).

Our investigation yielded three primary insights. First, the conformational search identified a unique “self-wrapping” mechanism in the most stable conformer structure (EA19), where oligoethylene glycol side chains fold inward to lock the conjugated backbone in a planar, thermodynamically robust configuration. Second, MD simulations revealed that high doping levels (75%) induce a cooperative electrostatic effect, leading to the strong confinement of counter-ions around the backbone, in sharp contrast to the diffuse ion hopping observed at lower doping (25%). Finally, and most critically, our electronic structure analysis demonstrated that this dense ionic environment acts as a double-edged sword. While high doping provides necessary charge carriers, the resulting electrostatic landscape heterogeneity significantly broadens the distribution of electronic coupling values (σ = 27.3 mHartree), introducing severe energetic disorder even in the absence of backbone torsional defects.

These findings highlight a fundamental trade-off in OMIEC design: maximizing carrier density inevitably complicates the local electrostatic landscape. Consequently, future strategies for optimizing OMIEC performance must go beyond simple backbone planarization and focus on electrostatic engineering—specifically, controlling the spatial arrangement of ions via precise side-chain architecture to mitigate environmental screening effects. The protocol established herein provides a powerful theoretical tool for guiding such rational design.

## Data Availability

The original contributions presented in this study are included in the article. Further inquiries can be directed to the corresponding author. The Python automation scripts, input templates, and data processing tools used in this study are available in the GitHub repository at https://github.com/gaozhanglei321/LAMMPS-Automation-Script (accessed on 23 February 2026).

## Supplementary Materials

The following supporting information can be downloaded at https://www.mdpi.com/article/10.3390/molecules31050774/s1, Additional figures supporting the structural analysis and convergence verification (Figures S1–S3) are provided.
