# Supplementary material for: Impact of Electrostatic Disorder on Intramolecular Electronic Coupling in Organic Mixed Ionic–Electronic Conductors: A Combined GRRM, MD, and QM/MM-CDFT Study
Source: Molecules. 2026 Feb 25;31(5):774. doi: 10.3390/molecules31050774 (PMC12986055; doi:10.3390/molecules31050774)
Supplement: Supplementary file 1 [file molecules-31-00774-s001.zip › molecules-4161715-supplementary.pdf]

Impact of Electrostatic Disorder on Intramolecular Electronic Coupling in Organic Mixed Ionic–Electronic Conductors: A Combined GRRM, MD, and QM/MM-CDFT Study

Zhanglei Gao [1†], Bowen Xiao [1†] , Naoki Kishimoto [2,*] and Takahiro Murashima [3]

1 Department of Chemistry, Graduate School of Science, Tohoku University, Aramaki, Aoba-ku, Sendai 980-8578, Japan; gao.zhanglei.p2@dc.tohoku.ac.jp
2 Institute for Excellence in Higher Education, Tohoku Universit 41 Kawauchi, Aoba-ku, Sendai 980-8576; kishimoto@tohoku.ac.jp
3 Department of Physics, Graduate School of Science, Tohoku University, 6-3 Aramaki Aza-Aoba, Aoba-ku, Sendai 980-8578, Japan; murasima@tohoku.ac.jp
* Correspondence: kishimoto@tohoku.ac.jp
† These authors contributed equally to this work.

Supporting Information

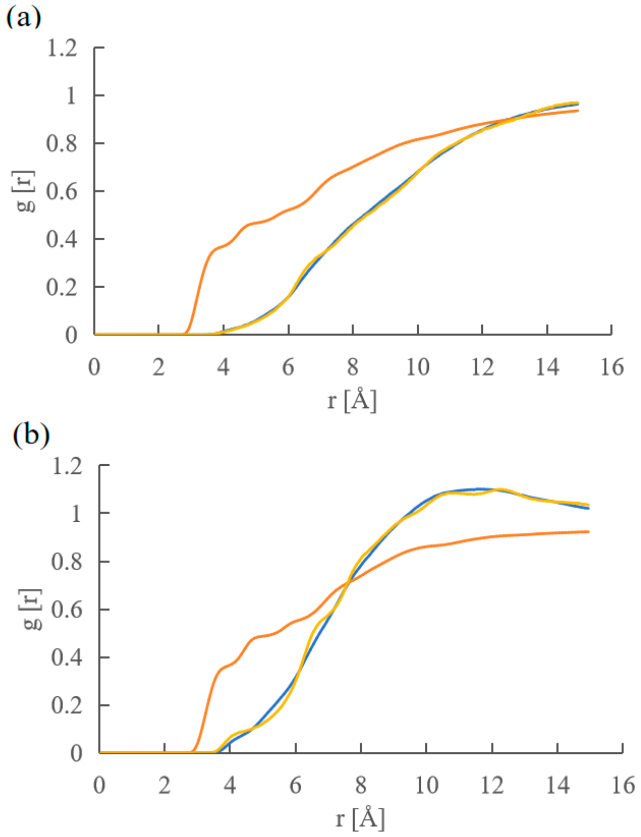

Figure S1. Comparison of radial distribution profiles for key interaction pairs in the (a) 25% and (b) 75% doping systems. The curves represent the spatial correlations between the polymer backbone carbon and chloride ions (blue, main_C–Cl^-), the backbone carbon and water oxygen atoms (orange, main_C–WAT_O), and the thiophene sulfur and chloride ions (yellow, S–Cl^-). The distinct profiles between the two doping levels further support the observation of enhanced ion condensation in the highly doped system.

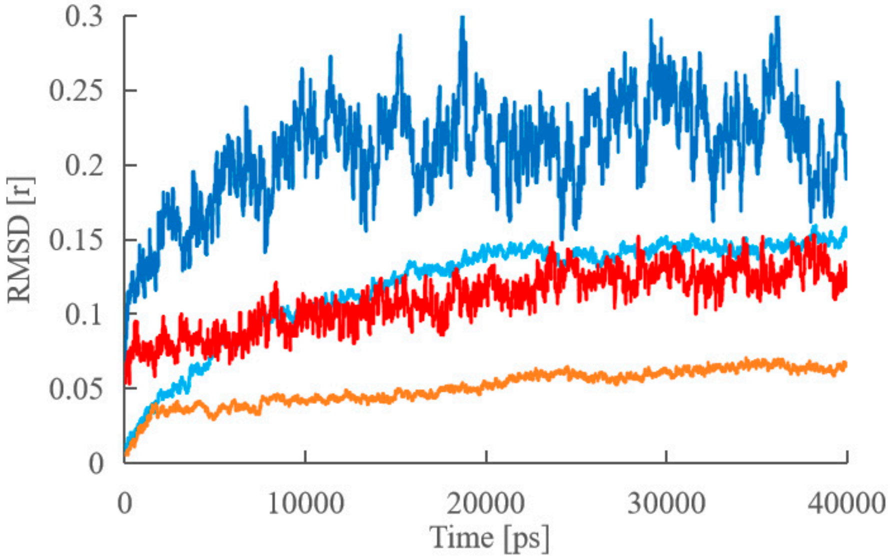

Figure S2. Time-dependent RMSD profiles of the radial distribution functions (RDFs) characterizing the structural convergence of the solvation shell and ion-polymer interactions. The plot displays the RMSD evolution for the pairs of polymer backbone carbon and water oxygen (main_C-WAT_O), and thiophene sulfur and chloride ions (S-Cl^-). The blue (S-Cl^-) and light blue (main_C-WAT_O) lines represent the 25% doping system, while the red (S-Cl^-) and orange (main_C-WAT_O) lines correspond to the 75% doping system.

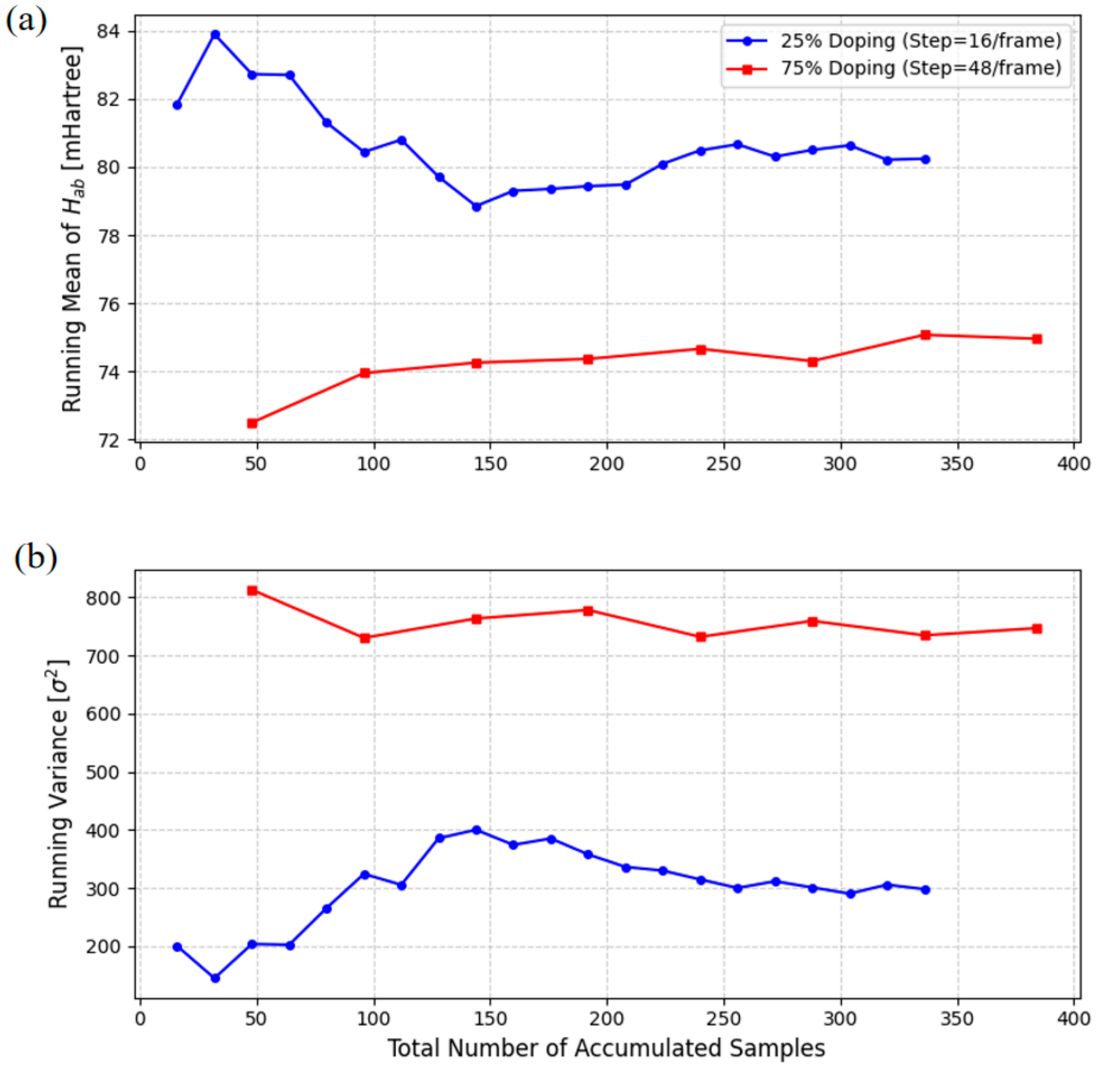

Figure S3. Convergence verification of the statistical descriptors for intrachain electronic coupling (H_{ab}). (a) Running mean [mHartree] and (b) running variance [σ^2] of H_{ab} calculated as a function of the total number of accumulated samples for the 25% (blue) and 75% (red) doping systems. The data points represent cumulative statistics calculated by sequentially adding MD snapshots (16 molecules/frame for 25% doping and 48 molecules/frame for 75% doping). Both descriptors exhibit rapid convergence and stabilization within the sampled dataset range (N=336 for 25% and N=384 for 75%), confirming statistical reliability.
